# Regulation of tracheal antimicrobial peptide gene expression in airway epithelial cells of cattle

**DOI:** 10.1186/s13567-016-0329-x

**Published:** 2016-03-17

**Authors:** Khaled Taha-Abdelaziz, Leanna Wyer, Lesley Berghuis, Laura L. Bassel, Mary Ellen Clark, Jeff L. Caswell

**Affiliations:** Department of Pathobiology, University of Guelph, Guelph, ON Canada; Department of Pathology, Faculty of Veterinary Medicine, Beni-Suef University, Beni-Suef, Egypt

## Abstract

β-defensins are an important element of the mucosal innate immune response against bacterial pathogens. Tracheal antimicrobial peptide (TAP) has microbicidal activity against the bacteria that cause bovine respiratory disease, and its expression in tracheal epithelial cells is upregulated by bacterial products including lipopolysaccharide (LPS, a TLR4 agonist), Pam3CSK4 (an agonist of Toll-like receptor 2/1), and interleukin (IL)-17A. The objectives of this study were to identify the signalling pathway by which LPS, Pam3CSK4 and IL-17A induce TAP gene expression, and to determine the effect of glucocorticoid as a model of stress on this epithelial innate immune response. In primary cultures of bovine tracheal epithelial cells (bTEC), LPS, Pam3CSK4 and IL-17A each stimulated TAP gene expression. This effect was abrogated by caffeic acid phenylester (CAPE), an inhibitor of NF-κB. Similarly, western analysis showed that LPS, Pam3CSK4 and IL-17A each induced translocation of NF-κB p65 from the cytoplasm to the nucleus, but pre-treatment with CAPE inhibited this response. Finally, pre-treatment of bTEC with the glucocorticoid dexamethasone abolished the stimulatory effect of LPS, Pam3CSK4 and IL-17A on upregulation of TAP gene expression. These findings indicate that NF-κB activation is necessary for induction of TAP gene expression by LPS (a TLR4 agonist), Pam3CSK4 (a TLR2/1 agonist), or IL-17A. Furthermore, this stimulatory response is inhibited by glucocorticoid, suggesting this as one mechanism by which stress increases the risk of bacterial pneumonia. These findings have implications for understanding the pathogenesis of stress-associated bacterial pneumonia, and for developing methods to stimulate innate immune responses in the respiratory tract of cattle.

## Introduction

Tracheal antimicrobial peptide (TAP) is a β-defensin produced by airway epithelial cells that has direct bactericidal activity against bacterial pathogens including those that cause respiratory disease in cattle [1–3]. Other defensins also have immunomodulatory functions that may contribute to respiratory health [4, 5] although such a role has not been reported for TAP. TAP gene expression is highly upregulated following exposure to inhaled bacteria or LPS. Thus, activation of TAP gene expression by Gram negative bacteria such as *Mannheimia haemolytica* represents an inducible mechanism of innate defence in the respiratory tract of cattle.

Risk factors for bovine respiratory disease are widely recognized and include the stresses of weaning, transportation, castration and inclement weather conditions, as well as viral infections, all of which occur at the time calves are removed from their dams and enter feedlots. These predisposing factors interfere with innate immune responses, alter bacterial populations in the nasal cavity, and are associated with increased number of bacteria reaching the lung [6]. Tracheal antimicrobial peptide is among the innate respiratory defences that are dysregulated by the effects of stress and glucocorticoid [7], viral infection including bovine viral diarrhea virus [8], and pollutants such as vanadium oxide in diesel exhaust [9]. Specifically, glucocorticoid and bovine viral diarrhea viral infection do not affect baseline expression of TAP in bTEC, but suppress the stimulatory effect of LPS both in vitro and in vivo. These findings suggest a mechanism by which stress and viral infection suppress innate defences in the respiratory tract and predispose to bacterial pneumonia.

Knowledge of these specific mechanisms by which respiratory defences fail suggests an opportunity to stimulate innate immune responses in the respiratory tract during times of susceptibility to pneumonia. The observation of a somewhat delayed effect of LPS, with peak effect at 16 h of stimulation [7, 10], prompted us to evaluate other agonists. We recently identified earlier induction of TAP gene expression in primary cultures of bovine tracheal epithelial cells (bTEC) following stimulation with agonists of TLR2/1 (Pam3CSK4) and IL-17A receptor (IL-17A) [10]. Greater understanding of the mechanisms of these innate immune responses may be of value not only for understanding pathogenesis, but also for development of novel methods to prevent disease. Thus, the objectives of this study were to identify the signalling pathway by which LPS, Pam3CSK4, and IL-17A upregulate TAP gene expression, and to determine whether this stimulatory pathway is similarly inhibited by glucocorticoid.

## Materials and methods

### Cell culture

Primary cultures of bovine tracheal epithelial cells (bTEC) were established and stimulated with agonists as previously described [10]. Briefly, bTEC were obtained from healthy market-weight beef cattle at slaughter, and a different donor was used for each experiment. Cells were grown to 80–90% confluency on collagen-coated plates in supplemented Dulbecco’s modified Eagle’s and Ham’s F-12 medium (DMEM/F12) containing 5% fetal bovine serum. Triplicate cell cultures supplemented with DMEM/F12 without serum were unstimulated, or stimulated with 0.1 μg/mL LPS (Sigma Aldrich, MO, USA, L9143), 1 μg/mL Pam3CSK4 (Invivogen, San Diego, CA, USA, t1rl-pms), or 316 ng/mL IL-17A (Kingfisher Biotech, St. Paul, MN, USA, RP0056B-005) for 16 h. The doses and time were optimized in a previous study [10].

To investigate the mechanism by which LPS, Pam3CSK4 and IL-17A induce TAP gene expression, bTEC were pre-treated for 2 h with 0.5–10 μM caffeic acid phenylester (CAPE; Calbiochem, San Diego, USA), an inhibitor of NF-κB signalling, before stimulation with LPS, Pam3CSK4 or IL-17A as described above. To investigate the role of c-Jun N-terminal kinase (JNK) in TLR2/1-mediated signalling, cells were treated for 2 h with 10–25 μM of the JNK inhibitor SP600125 (Sigma-Aldrich, USA), prior to stimulation with Pam3CSK4. The findings were confirmed by repeating the study with bTEC from a different animal.

To measure the effect of corticosteroid on inducible expression of TAP, bTEC were treated in triplicate with 10^−6^ M dexamethasone sodium phosphate (Vétoquinol, Lavaltrie, QC, Canada) in supplemented DMEM/F12 for 24 h before 16-h stimulation with agonists (a total of 40 h of dexamethasone treatment). This dose and time had been optimized in a previous study [7]. The findings were confirmed by repeating the study with bTEC from a different animal. To measure cell viability and cytotoxicity of the treatments, lactate dehydrogenase (LDH) activity was measured in supernatants collected at the end of the experiments, following the manufacturer’s instructions (Cytotoxicity Detection Kit, Roche Diagnostics, Mannheim, Germany).

RNA extraction and cDNA synthesis were carried out as previously described [10]. Briefly, total RNA was extracted (RNEasy® Mini kit, Qiagen), treated with DNase I, eluted in RNase-free water, and 100 ng of total RNA was reverse transcribed to cDNA using Superscript III. Real-time reverse transcription quantitative PCR was conducted using LightCycler 480 technology (Roche Diagnostics) to quantify relative gene expression between stimulated and non-stimulated as well as pre-treated bTEC. Gene expression of TAP was measured relative to the reference gene glyceraldehyde-3-phosphate dehydrogenase (GAPDH) as previously described [10]. Threshold cycles for both TAP and the reference gene (GAPDH) calculated by the LightCycler 480 software (Roche Diagnostics) were normalized to a calibrator, and data of technical duplicate samples were averaged.

### Western analysis of NF-κB p65

Localization of NF-κB p65 to the cytoplasm and nucleus was analyzed by western blot. For preparation of cytosolic and nuclear protein fractions, stimulated bTEC were washed with cold PBS and lysed as described [11, 12]. Cells were harvested by scraping into lysis buffer containing 10 mM HEPES pH 7.9, 1.5 mM MgCl_2_, 10 mM KCl, 0.25% v/v noident P-40, 0.5 mM phenylmethylsulfonyl fluoride (PMSF), and 0.5 mM dithiothreitol (DTT); then incubated for 20 min on ice. Cells were centrifuged at 12 000 ×* g* for 15 s at 4 °C and supernatant (the cytoplasmic fraction) was collected. The remaining pellet was lysed by adding 15 μL of 20 mM HEPES pH 7.9, 1.5 mM MgCl_2_, 420 mM NaCl, 0.5 mM DTT, 25% v/v glycerol, and 0.5 mM PMSF, incubating for 20 min on ice, then centrifuged at 12 000 ×* g* for 60 s at 4 °C. The supernatant (the nuclear fraction) was collected and 60 μL of 20 mM HEPES pH 7.9, 50 mM KCl, 0.5 mM DTT, 0.2 mM EDTA, and 0.5 mM PMSF was added. The protein concentrations of the cytoplasmic and nuclear extracts were measured by spectrophotometry (Nanodrop 2000, Thermo Scientific, Wilmington, DE, USA).

Samples of nuclear or cytoplasmic extracts (20 µg protein) or positive control NF-κB p65-transfected cell lysate (10 µg protein; Santa Cruz Biotechnology, USA) were separated by 10% SDS-PAGE at 120 V for 2 h and then blotted on a PVDF membrane at 90 V for 1 h. The amount of protein loaded was initially optimized based on Coomassie blue-stained SDS-PAGE. Membranes were blocked by incubation with 5% skim milk in Tris PBS-0.1% Tween for 2 h with rocking then washed several times for 30 min, followed by incubation with rabbit anti-NF-kB p65 antibody (1:200, Santa Cruz Biotechnology, USA) for 2 h with rocking at room temperature. Membranes were washed for 30 min before incubation with goat anti-rabbit HRP (1:4000; Dako, Denmark) for 30 min with rocking at room temperature. Membranes were washed and then incubated with enhanced chemiluminescence reagent for 1 min. The bands were visualized and photographed.

### Statistical analysis

Real-time RT-PCR data (normalized ratios of TAP:GAPDH) were log-transformed if needed to normalize the distribution, then analyzed by one- or two-way ANOVA test (Graphpad prism V5.0, Graphpad software, San Diego, CA, USA), and considered significant at *P* < 0.05. Graphical data are presented as mean ± standard error of the mean (SEM).

## Results

### Role of NF-κB and JNK signalling in inducible TAP gene expression in bTEC

**Figure 1:**
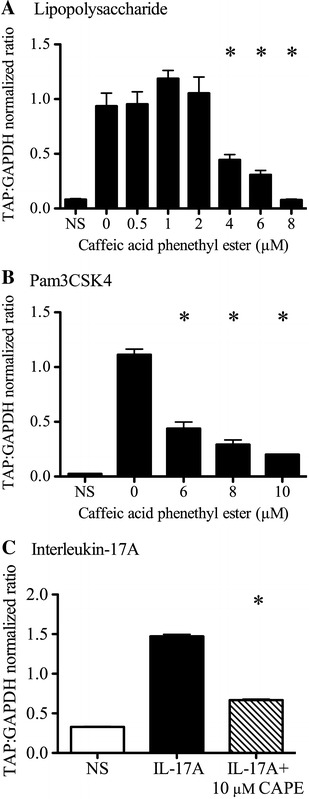
**Effect of the NF-κB inhibitor caffeic acid phenylester (CAPE) on induction of TAP gene expression by LPS, Pam3CSK4 and IL-17A.** Stimulation of bovine tracheal epithelial cells (bTEC) with 0.1 µg/mL LPS (panel** A**), 1 µg/mL Pam3CSK4 (panel** B**), or 316 ng/mL IL-17A (panel** C**) induced significantly greater TAP gene expression than in non-stimulated cells (NS). Pre-treatment with CAPE for 2 h before stimulation inhibited each of these responses. Asterisks indicate significant effects of CAPE treatment compared to no CAPE, *P* < 0.0001, one-way ANOVA. The data are mean ± SEM of technical triplicates, and the findings were similar when the experiments were repeated using cells from different animals.

Treatment of bTEC with LPS, Pam3CSK4, or IL-17A resulted in significant induction of TAP gene expression compared to non-stimulated cells. Pre-treatment with CAPE (an inhibitor of NF-κB activation) suppressed this stimulatory effect for all three agonists: LPS, Pam3CSK4, and IL-17A (Figure 1; *P* < 0.0001 for each agonist, one-way ANOVA with Tukey’s post-test). At maximal effect using 8 μM CAPE, TAP gene expression in LPS-treated cells was not significantly different from unstimulated cells (Figure 1A). Treatment of bTEC with 6–10 μM CAPE resulted in dose-dependent inhibition of Pam3CSK4-induced TAP gene expression (Figure 1B), with 84% reduction in TAP gene expression using 10 μM CAPE. Treatment with 10 μM CAPE reduced IL-17A-induced TAP gene expression by 55% (Figure 1C).

**Figure 2:**
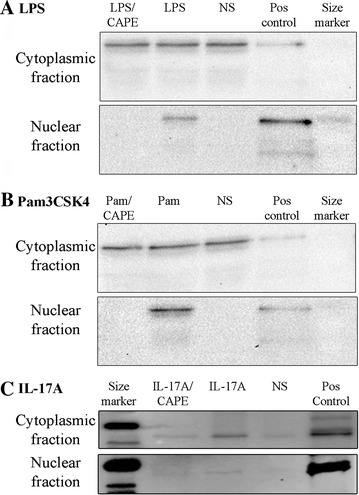
**Western analysis of NF-kB p65 in nuclear and cytoplasmic extracts of bTEC stimulated with LPS, Pam3CSK4 or IL-17A.** NF-kB p65 was localized to the cytoplasm of non-stimulated cells (NS), but showed translocation to the nucleus following treatment of the cells with either LPS (panel** A**), Pam3CSK4 (Pam, panel** B**) or IL-17A (panel** C**). However, pre-treating the cells with the NF-κB inhibitor CAPE (10 μM) prior to exposure to agonists fully abrogated nuclear translocation of NF-kB p65 in the studies involving LPS and Pam3CSK4 (panels** A** and** B**), and partially in the studies involving IL-17A (panel** C**). Pos control: positive control, NF-kB p65. The findings were similar when the experiments were repeated using cells from different animals.

Cytoplasmic and nuclear NF-κB p65 were examined by western blot analysis of bTEC to further confirm the involvement of NF-κB in induction of TAP gene expression by LPS, Pam3CSK4, or IL-17A. In unstimulated cells, NF-κB p65 was detected in the cytoplasmic but not in the nuclear fractions (Figure 2). In bTEC that were stimulated for 16 h with 0.1 µg/mL LPS, NF-κB p65 was detected in both the nuclear and the cytoplasmic fractions. In contrast, in bTEC treated with 10 µM CAPE prior to stimulation with LPS, NF-κB p65 was detected only in the cytoplasmic fractions (Figure 2A). Similarly, in bTEC stimulated for 16 h with 1 µg/mL Pam3CSK4, NF-κB p65 was detected in both the nuclear and the cytoplasmic fractions, whereas NF-κB p65 was detected only in the cytoplasmic fractions in bTEC treated with 10 µM CAPE prior to stimulation with Pam3CSK4 (Figure 2B). Finally, NF-κB p65 was detected in the nuclear fraction of bTEC stimulated for 16 h with 316 ng/mL IL-17A, whereas treatment with 10 µM CAPE prior to stimulation with IL-17A partially inhibited this effect (Figure 2C). These findings show that LPS, Pam3CSK4, or IL-17A induce translocation of NF-κB p65 from the cytoplasm to the nucleus, with inhibition of this effect by CAPE.

**Figure 3:**
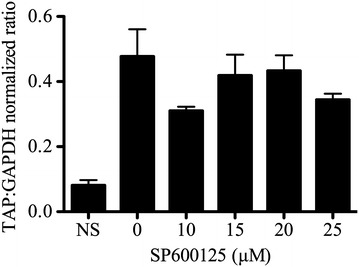
**Effect of SP600125 on** **Pam3CSK4-induced TAP gene expression.** Pre-treatment of bTEC with various concentrations of the JNK inhibitor SP600125 had no significant effect on Pam3CSK4-induced TAP gene expression (*P* > 0.05, one-way ANOVA, *n* = 3 per group).

The role of JNK in Pam3CSK4-induced TAP gene expression was evaluated using the JNK inhibitor SP600125. Treatment of bTEC with different doses (10–25 µM) of SP600125 had no significant effect (*P* > 0.05, one-way ANOVA) on Pam3CSK4-induced stimulation of TAP gene expression (Figure 3).

RT-PCR crossing points of GAPDH mRNA were not affected by treatment with LPS, Pam3CSK4, IL-17A, CAPE or SP600125. Based on measurement of LDH in cell culture supernatants, cell viability was greater than 98% following exposure to either CAPE or SP600125, and morphologic evidence of cytotoxicity was not observed.

### Effect of corticosteroids on upregulation of TAP gene expression

**Figure 4:**
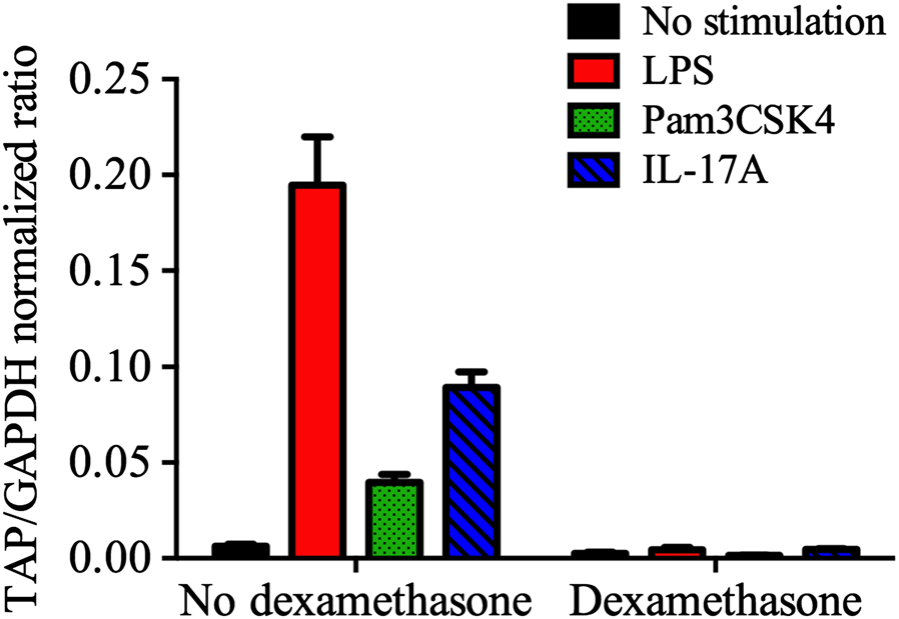
**Effect of dexamethasone on LPS-, Pam3CSK4- and IL-17A-induced up-regulation of TAP mRNA.** Bovine tracheal epithelial cells were grown in the presence or absence of 10^−6^ M dexamethasone for 24 h. LPS (0.1 μg/mL), Pam3CSK4 (1 μg/mL) or IL-17A (316 ng/mL) were added for 16 h. Each of these agonists significantly upregulated TAP gene expression relative to the reference gene GAPDH (*P* < 0.001, two-way ANOVA of log-transformed data, Tukey’s post-test). The LPS-, Pam3CSK4- and IL-17A-induced upregulation were each abrogated by pre-treatment with dexamethasone (*P* < 0.001, two-way ANOVA of log-transformed data, Tukey’s post-test, *n* = 3 per group). Repetition of the experiment using cells from a different animal yielded similar findings.

LPS, Pam3CSK4 and IL-17A each significantly (*P* < 0.05) induced TAP gene expression in bTEC compared to the non-stimulated controls. Pre-treatment with dexamethasone abrogated the stimulatory effect of either LPS, Pam3CSK4 or IL-17A (*P* < 0.05, 1-way ANOVA), to levels equal to that of the non-stimulated cells (Figure 4). LDH release (cytotoxicity) was not observed as a consequence of treatment with LPS, Pam3SCK4, IL-17A, or dexamethasone (Figure 4). The findings were not significantly different when agonists were added to medium containing 5% fetal bovine serum, rather than the serum-free medium used above (data not shown).

## Discussion

Innate immune responses are normally effective in protecting the lung, but are susceptible to failure as a result of stress and viral infection. Thus, strategies to restore these responses may be useful for disease prevention. The agonists LPS, Pam3CSK4, and IL-17A induce rapid and strong induction of TAP gene expression in bTEC. The present study indicates NF-κB signalling as a necessary pathway for this effect. However, the stimulatory effects of LPS, Pam3CSK4, and IL-17A on TAP gene expression were all inhibited by dexamethasone treatment, showing that the suppressive effects of glucocorticoids and possibly of stress on this epithelial innate immune response are not specific for one pathway of cell activation.

CAPE is an inhibitor of IkBα phosphorylation and of subsequent activation and nuclear translocation of NF-kB [13, 14]. Treatment of bTEC with CAPE resulted in abrogation of the stimulatory effects of LPS and Pam3CSK4, indicating a key role for NF-κB activation in development of this innate immune response. For IL-17A, the response was not completely abolished by CAPE, suggesting a possible involvement of other signalling pathways in IL-17A-induced TAP gene expression. For example, a prior study identified a role for the transcription factor Oct-1 in phorbol myristate acetate-stimulated induction of TAP gene expression in a bovine mammary epithelial cell line [15]. Thus, further studies are needed to more fully understand the regulation of IL-17A—mediated induction of TAP gene expression. We considered the possibility of CAPE-induced cytotoxicity as a potential reason for reduction in TAP gene expression. However, this is excluded by the fact that expression of the reporter gene GAPDH was not similarly inhibited, LDH was not detected in supernatants, and morphologic evidence of cytotoxicity was not observed.

Western blot analysis of NF-κB p65 provided an alternative experimental approach to confirm the above findings. Treatment of bTEC with LPS, Pam3CSK4, or IL-17A resulted in translocation of NF-kB p65 from cytoplasm to nucleus, indicating that the NF-kB signalling pathway is induced by these agonists. For Pam3CSK4 and LPS, this effect was completely blocked by prior treatment with CAPE, with all NF-κB p65 remaining in the cytoplasm. In contrast, CAPE pre-treatment reduced but did not completely abrogate NF-κB translocation to the nucleus in the experiments involving stimulation with IL-17A. These findings confirm the above-noted effects of CAPE on this signal transduction pathway. Earlier studies identified an NF-κB-binding site in the promoter region of the TAP gene [15], and suggested a role of NF-κB in LPS-induced TAP gene expression by identifying that NF-κB and NF IL-6 binding sites in the region of the TAP gene were important for gene expression based on a luciferase reporter assay [16]. The present study extends this genetic approach to confirm the importance of NF-κB using two functional assays in the target cells of interest, and further identifies an important role of NF-κB in mediating the stimulatory effect of Pam3CSK4 and IL-17A.

The JNK signalling pathway was investigated as an alternative pathway for Pam3CSK4-induced stimulation of TAP gene expression, because this pathway is necessary for induction of human β-defensins -2 and -3 by pathogenic bacteria and for TLR2/1-induced production of proinflammatory cytokines [17–19]. However, the JNK inhibitor had no significant effect on Pam3CSK4-induced TAP gene expression, implying that this pathway is not necessary for this effect. Overall, these findings are consistent with a necessary role for NF-κB in upregulation of TAP gene expression by LPS, Pam3CSK4, or IL-17A, and suggest that strategies to stimulate NF-κB signalling pathways may be of value for enhancing production of TAP by airway epithelial cells.

A second goal of the study was to investigate the impact of glucocorticoids on LPS-, Pam3CSK4- and IL-17A-mediated up-regulation of TAP gene expression, as a mechanism by which stress modulates the innate respiratory defenses. Dexamethasone had no effect on the very low constitutive level of TAP gene expression in bTEC, but inhibited the up-regulation of TAP gene expression following stimulation with LPS, Pam3CSK4, or IL-17A. As above, cytotoxicity was excluded as a reason for this inhibitory effect.

These findings indicate that the previously discovered inhibitory effects of glucocorticoid on TAP gene expression [7] are not limited to LPS stimulation acting through TLR4, but that similar inhibitory effects occur when Pam3CSK4 or IL-17A are used as agonists. Although additional work is needed to identify precise mechanisms by which dexamethasone exerts these effects, these findings clearly show that the effect is not unique to TLR4 expression or function. Instead, dexamethasone may have a more general suppressive effect on, for example, the expression of a broader range of cell surface receptors, I-κB kinase function, expression or stability of I-κB, or the promoter region of the TAP gene [20].

The use of primary cell cultures rather than cell lines is advantageous to ensure the findings of this study have in vivo relevance [21]. It is acknowledged that primary cultures of bTEC overlook the complex interactions that occur in vivo in the respiratory tract such as, for example, effects of glucocorticoids on leukocytes that subsequently affect epithelial cell responses. Nonetheless, this strategy is favourable as it focuses on responses of bTEC to understand the biology of these epithelial cell responses during stress and inflammation.

Together, these findings extend our knowledge of how innate immune responses of the respiratory tract are induced, and how they are susceptible to failure under conditions of glucocorticoid excess. Specifically, this study establishes NF-κB as a necessary mediator of LPS-, Pam3CSK4-, or IL-17A-induced upregulation of TAP gene expression, and shows that corticosteroid abrogates the immunostimulatory effects of TLR2/1, TLR4 and IL-17AR. These findings have implications with respect to developing methods to restore innate immune responses in stressed cattle.

## Abbreviations

bTEC: bovine tracheal epithelial cells
CAPE: caffeic acid phenylester
DMEM/F12: Dulbecco’s modified Eagle’s and Ham’s F-12 medium
DTT: dithiothreitol
GAPDH: glyceraldehyde-3-phosphate dehydrogenase
IL: interleukin
JNK: c-Jun N-terminal kinase
LDH: lactate dehydrogenase
LPS: lipopolysaccharide
PMSF: phenylmethylsulfonyl fluoride
SEM: standard error of the mean
TAP: tracheal antimicrobial peptide
